# Investigating Liposome Membrane Properties: Insights from Langmuir Monolayer Studies in the Corona Protein Environment

**DOI:** 10.1021/acs.jpcb.5c03676

**Published:** 2025-08-19

**Authors:** Michalina Zaborowska-Mazurkiewicz, Natalia Kraśkiewicz, Piotr Sekuła, Renata Bilewicz

**Affiliations:** 49605 University of Warsaw, Faculty of Chemistry, Pasteura 1, 02093 Warsaw, Poland

## Abstract

Most nanoparticles approved for medical applications are liposome formulations. In the environment of biological fluids, the corona protein was found to alter both the surface of the liposome and its size and zeta potential. As a result, it can also affect the stability of the carrier and its ability to hold a drug and release it sustainably into the cell. The key element of novelty of this research is the introduction of the Langmuir–Blodgett technique together with imaging microscopy to study the interactions between the monolayer corresponding to one liposome leaflet and the corona protein. We applied this approach to study the properties of individual lipid monolayers, which constitute the bilayers of PEGylated or non-PEGylated liposomes exposed to human serum albumin solutions. The thermodynamic parameters obtained from these measurements reflect changes in the properties of the lipid layer under the influence of the corona protein. Polarization modulation infrared reflection absorption spectroscopy (PM-IRRAS) spectra visualized the differences of these interactions, depending on the composition of the lipid layer. Liposome properties were also characterized in the PBS solution before and after incubation in human serum albumin solutions. The antirheumatic drug, iguratimod, was chosen as the cargo of the liposomes, and changes in its release kinetics in the presence and absence of the corona protein confirmed the advantages of the selected PEGylated lipid carrier.

## Graphical abstract

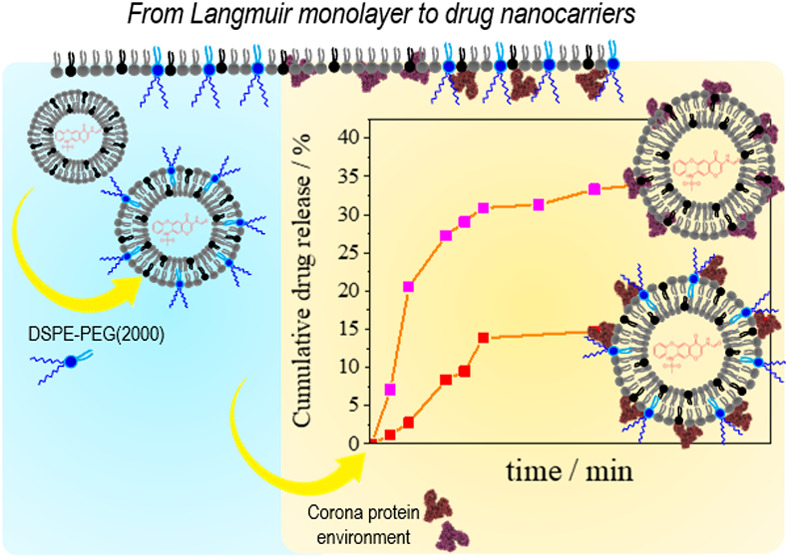

## 1 Introduction

Liposomes predominate among nanoparticles used for drug delivery, and active research continues to explore the most suitable composition of these carriers to enhance the bioavailability of the drug.^1–3^ They were found to reduce side effects and improve sustainable and better-targeted delivery. The factors to consider when designing the carrier are nanoparticle stability, interactions with the biological environment, and suitability for encapsulating a specific drug, taking into account the drug’s stability, solubility, hydrophobicity, and charge.^4,5^ Specifically, this is related to the presence of proteins and other biomolecules in plasma, which readily interact with liposomes to form a corona protein on their surface.^6–9^ Possible effects of the corona protein include liposome shrinkage, changes in zeta potential, disruption of the lipid bilayer, and protein-mediated aggregation.^7^ Understanding these interactions is a prerequisite for developing safe and sustainable formulations that are useful for clinical applications. Because the interaction with proteins and the formation of a more or less dense corona controls the properties of the carriers, we focus on two aspects of the liposome’s contact with corona proteins: their interactions and the choice of a suitable modification for the liposome’s external layer.

In this study, human serum albumin (HSA) was used as a simplified model to investigate the initial interactions between liposomes and a representative plasma protein. In human blood plasma, the total protein content is typically 60–80 mg/mL (approximately 6–8% w/v), with albumin constituting about 35–50 mg/mL. Under such physiological conditions, drug delivery systems are exposed to a highly dynamic and complex mixture of proteins. Higher protein concentrations can lead to more extensive and competitive adsorption processes (e.g., the Vroman effect),^10^ resulting in the formation of a complex and evolving protein corona. The use of a lower HSA concentration enabled us to isolate and analyze specific interactions under well-defined conditions, future studies will include full serum or plasma to more closely mimic the in vivo environment and to assess the robustness of our observations under physiological conditions.^11^

The PEGylation of the interfaces of lipid nanoparticles was reported by Angelov et al.^12^ to impart membrane flexibility and provide steric stabilization to the nanostructured lipid carriers. PEGylation has been repeatedly reported to prolong liposome circulation and improve their uptake by diseased cells.^13^ There is an ongoing discussion of the role of PEG in determining the amount of corona protein that can adsorb onto the liposome. A decrease in binding has usually been reported.^12–15^ However, examples have also been provided showing that proteins exhibit higher affinity for PEGylated liposomes than for their non-PEGylated counterparts. The DSPE-PEG(2000) used in our work is negatively charged and may partially neutralize the charge of the positively charged liposome components, thereby decreasing the liposome’s affinity toward albumin. According to Thakur et al.,^14^ the degree of HSA penetration into lipid bilayers depends on the lipid saturation. In the case of unsaturated lipids, such as DOPC or POPC, the interaction is limited to the bilayer surface, whereas with saturated lipids, such as DMPC or DPPC, the interaction is more extensive. DDAB is a surfactant that increases liposome stability, as well as the extent of drug incorporation. Because of its positive charge, it enhances the affinity for cell membranes.^15^

The cargo of the liposome carrier in our study is iguratimod (Igu, Figure 1)—a small molecule immunomodulator and antirheumatic drug with remarkable effectiveness in the treatment of active rheumatoid arthritis.^16–19^ It inhibits various inflammatory cytokines and regulates the balance of immune cells and cytokines. Its osteoprotective action is related to the promotion of osteoblast differentiation and the inhibition of osteoclastogenesis.^20–22^ Iguratimod-encapsulating poly­(d,l-lactic-coglycolic acid) (PLGA-NPs) were demonstrated to induce human multiple myeloma cell death via reactive oxygen species and caspase-dependent signaling.^23^ Despite several benefits, enhanced ROS generation ultimately led to cell apoptosis. Therefore, it would be of significant importance to design alternative and more robust drug delivery carriers that provide sustained release of iguratimod in the environment of cell proteins.

**Figure 1:**
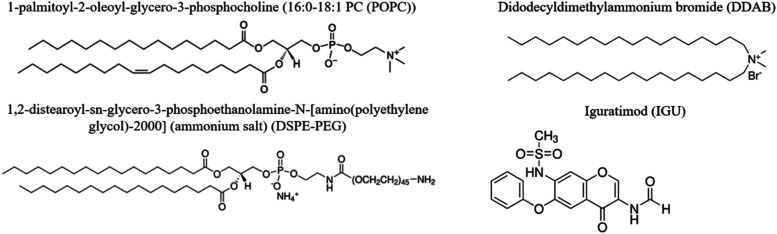
Chemical structures of chemicals used in the described experiments.

All of the above inspired us to study the influence of PEGylation on the single layers that make up the liposome, and a particularly suitable method for investigating the properties of monolayers and the incorporation of other molecules is the Langmuir method. Therefore, the properties of POPC/DDAB and POPC/DDAB (/DSPE-PEG (2000)) monolayers on the PBS subphase and the albumin-containing subphase were investigated using the Langmuir method supported by polarization modulation infrared reflection absorption spectroscopy (PM-IRRAS). These two carrier shell compositions (POPC/DDAB and POPC/DDAB/DSPE-PEG (2000)), (Figure 1) are compared and the contribution of PEGylation to the overall interaction of liposomes with human serum albumin is evaluated. The first component, POPC, is a common unsaturated component of biological membranes, so its presence in liposomes should increase the affinity of the liposome for cell membranes.^17^ The properties of liposomes as an iguratimod carrier were analyzed from the point of view of stability, degree of encapsulation, and release profile of the drug before and after incubation in human serum albumin solutions. Taking into account the difference that the Langmuir monolayer is planar, while the liposome is a curved structure, the advantages of the Langmuir methodology (2D membrane model) are clear. It allows probes of (i) molecular interactions between components within the monolayer, (ii) the effect of human serum albumin (HSA) on layer composition and organization, and (iii) the strength of drug–lipid interactions. On the contrary, the spherical (3D) liposome is the carrier used, allowing evaluation of the efficiency of drug loading, the stability of the system, and drug release properties, also under real biological conditions. Together, these two models, the 2D monolayer and the 3D liposome, provide reliable information on the complex interactions essential for the rational design of a liposomal drug delivery system.

## 2 Experimental Section

### 2.1 Materials

The reagents used in the experiments described here are as follows: 1-palmitoyl-2-oleoyl-*sn*-glycero-3-phosphocholine (POPC), a phospholipid containing 16:0 and 18:1 acyl chains; didodecyldimethylammonium bromide (DDAB); 1,2-distearoyl-*sn*-glycero-3-phosphoethanolamine-*N*-[amino­(poly­(ethylene glycol))-2000] (ammonium salt) (DSPE-PEG(2000)), used as stabilizer for carriers; and iguratimod (Igu). All reagents were purchased from Merck (Poland), except iguratimod, which was acquired through Trimen Chemicals (Poland). The reagents for liposome preparation were dissolved in high-purity solvents, specifically chloroform and methanol, while iguratimod was dissolved in dimethyl sulfoxide (DMSO, POCH, Gliwice, Poland). Two different systems were employed and compared as drug nanocarriers: POPC/DDAB in a molar ratio of 9/1 and POPC/DDAB/DSPE-PEG(2000) in a molar ratio of 8:1.5:0.5. Human serum albumin (HSA, Merck, Poland) was used to form a corona protein on the liposome surface and study the interactions between the components of the lipid bilayer with this protein. HSA was used in two concentrations: lower (0.1%) for studying the interactions of albumin with layer components (Langmuir method) and higher (1%) to create a corona protein around the liposome. The rationale for selecting relatively small concentrations of HSA, 0.1% and 1% (w/v) was to enable analysis of the influence of liposome curvature and membrane composition on albumin adsorption, while still allowing the observation of subtle effects related to the liposomal bilayer. These HSA concentrations were in a range common for similar studies.^24^ 0.01 M Phosphate buffer (PBS), pH 7.4, and 36.6 °C was used to mimic the physiological conditions and keep the pH constant.

### 2.2 Langmuir Monolayer Technique

The model lipid monolayers were prepared using a Langmuir apparatus consisting of hydrophilic movable barriers (with a compression rate of 10 mm/min) and a Teflon Langmuir trough (*A* = 243 cm^2^). The surface pressure recorder was a Wilhelmy plate (±0.1 mN/m) coupled to a Wilhelmy balance. Measurements were made at room temperature. Surface pressure – area per molecule (π – *A*) isotherms were recorded and the compressibility modulus (*C*_s_^–1^) describing the state of the monolayer was calculated using the equation

1 $$C_{s}^{-1}=-A{\left(\frac{\partial \pi }{\partial A}\right)}_{T}$$

Based on the value of *A* ^Exc^ (eq 3) for mixed monolayers, interactions between components can be characterized as attractive if *A* ^Exc^ < 0, or repulsive if *A* ^Exc^ > 0 than interactions in single component layers.

2 $$A_{123}^{\mathrm{id}}=A_{1}X_{1}+A_{2}X_{2}+A_{3}X_{3}$$

3 $$A^{\mathrm{Exc}}=A_{123}-A_{123}^{\mathrm{id}}$$

4 $$\%A=\frac{A_{1..N}^{id}-A_{1...N}}{A_{1..N}^{id}}\cdot 100\%$$

5 $$\Delta G^{M}=\Delta G^{\mathrm{Exc}}+\Delta G^{\mathrm{id}}$$

In the above eqs (eqs 2 and 3) *A*_123_^id^ is the theoretical total area per molecule in the monolayer, calculated on the basis of the area per molecule (*A*_i_) at a given surface pressure for a single-component monolayer, and *X*_i_ is the mole fraction of this component in the mixed layer. *A*_123_ is the area value obtained experimentally for the mixed monolayer. The quality of the mixing of the components in the layer and the thermodynamic stability are determined by the free energy of mixing, Δ*G* ^M^ (eq 5).

The compression/expansion of the monolayers allowed to calculate the free energy difference corresponding to the recorded hysteresis (Δ*G* ^hys^), the configurational entropy of hysteresis (Δ*S* ^hys^) and the enthalpy of hysteresis (Δ*H* ^hys^) in the monolayer experiments performed in the absence and presence of HSA^25,26^

6 $$\Delta G^{\mathrm{hys}}=\Delta G_{\exp}-\Delta G_{\mathrm{comp}}$$

7 $${\left[\Delta S_{\pi }^{\mathrm{hys}}=R\ln\frac{A_{\exp}}{A_{\mathrm{comp}}}\right]}_{\pi }$$

8 $$\Delta H^{\mathrm{hys}}=\Delta G^{\mathrm{hys}}+T\Delta S^{\mathrm{hys}}$$

The incorporation of HSA into the preformed monolayers (POPC/DDAB and POPC/DDAB/DSPE-PEG(2000)) was determined by recording the dependence of surface pressure, and area per molecule changes on time after the injection of 0.1% HSA.

### 2.3 Polarization Modulation Infrared Reflection Absorption Spectroscopy (PM-IRRAS)

The IR spectra of the monolayers at the air/water interface were recorded using the following methods. Nicolet iS50 FT-IR spectrometer (Thermo Scientific) with ZnSe photoelastic modulator (PEM was set to 1500 cm^–1^, 50 kHz) and liquid N_2_ cooled detector (MCT-A). Spectra were recorded at a constant surface pressure of 30 mN/m in the wavelength range of 4000–800 cm^–1^ (analyzed ranges from 3050 to 2750 cm^–1^ and 1750 to 1000 cm^–1^) with a resolution of 4 cm^–1^. The software that enabled the measurement and analysis of the spectra was Omnic Modulation. The final reference spectrum (*S*) is calculated as the ratio of the difference and the sum of the intensities of two polarizations (*I*_s_ and *I*_p_)­

9 $$S=\frac{I_{s}-I_{p}}{I_{s}+I_{p}}$$

The final spectrum presented in this publication (after background correction) is defined as

10 $$\Delta S=\frac{S_{\pi }}{S_{0}}$$

Each time, the background spectrum (pure subphase, *S*_0_) and the lipid layer (*S*_π_) were recorded, and the results presented are the effect of averaging at least three spectra.

### 2.4 Liposome Characterization

The mixtures of POPC/DDAB in a molar ratio of 9/1 and POPC/DDAB/DSPE-PEG(2000) in a molar ratio of 8:1.5:0.5 in organic solvents were prepared. Subsequent portions of the mixtures with a given composition were then transferred to Eppendorf vessels, and the organic solvents were evaporated in a stream of argon (with limited access to oxygen). In this way, a thin layer of lipid film was obtained, which was then placed in a desiccator for at least 2 h to remove residual solvents. To obtain the liposomes, the thin lipid film was hydrated by adding 1 mL of PBS buffer at a concentration of 0.01 M and pH 7.4 or a buffer with the addition of a drug at a given concentration (10^–5^ M). Hydration was carried out for 1.5 h in an ultrasound bath with heating set at 37 °C (above the phase transition temperature of the lipid mentioned, Tm, POPC *=* −2 °C). The liposomes were extruded through filters with a pore diameter of 100 nm. Hydrodynamic diameter (DLS) and zeta potential (ξ) measurements for lipid nanocarriers were performed at 36.6 ± 1 °C (as physiological temperature) using the Zetasizer Nano ZSP (Malvern Panalytical, Malvern, U.K.). To create the corona protein, liposomes (with and without the drug, iguratimod) were incubated in a solution of 1% human serum albumin (36.6 °C).

### 2.5 UV–vis Spectroscopy

The UV–vis spectrophotometric method was employed to quantify the concentration of free drug not bound to the lipid carrier and to evaluate the sustained drug release process from the carrier in various lipid environments. For this purpose, cuvettes were used to collect fractions from a Sephadex G-25 column (Cytiva, Danaher), which was used to separate liposomes from the unbound drug. Quantification was carried out comparing with the calibration curve, covering iguratimod concentrations ranging from 1·10^–8^ to 5·10^–5^ M. The characteristic wavelength to monitor the Igu concentration was 258 nm. Measurements were made using a Synergy LUX multimode reader (BioTek Inc.) spectrophotometer. A calibration curve for the drug used was used to determine the encapsulation efficiency (EE%) based on the following equation: where the concentration values were used for the drug added with the hydrating solution (*C*_total_) and those calculated for the free drug on the basis of the calibration curve (*C*_free_)­

13 $$\mathrm{EE}\left(\%\right)=\frac{C_{\mathrm{total}}-C_{\mathrm{free}}}{C_{\mathrm{total}}}\cdot 100\%$$

where the concentration values were used for the drug added with the hydrating solution (*C*_total_) and the one calculated for the free drug based on the calibration curve (*C*_free_).

The drug release process from binary and ternary liposomes in the presence and absence of the HSA corona protein was compared. A sample of isolated liposomes was placed in a tube with a diffusion membrane (Pur-A-Lyzer), which was then immersed in a solution to which the drug was intended to be released by a concentration gradient. This solution was stirred using a magnetic stirrer and the entire system was incubated in a water bath at 36.6 °C. The external solution used was a phosphate buffer at pH 7.4 (*C* = 0.01 M). The concentration of iguratimod (Igu) was measured by UV–vis spectrophotometry at a wavelength of 258 nm, as previously described.

Since the release of therapeutics from carriers depends on different factors (including carierr physicochemical properties, drug type, and environmental conditions), several mathematical drug release models were analyzed. To fit the drug release kinetics model, we considered the zero- and first-order release kinetics, Higuchi, and Korsmeyer-Peppas models.^27–29^ In the zero-order model, the drug is released at a constant rate, regardless of its concentration. The first-order model assumes that the release rate decreases as the drug concentration decreases, while the Higuchi model describes the drug diffusion process as being dependent on the square root of time. The Korsmeyer-Peppas model allows determining which mechanism is preferred in the given system.^30^

## 3 Results and Discussion

### 3.1 Comparison of Langmuir Monolayers Composed of POPC/DDAB and POPC/DDAB/DSPE-PEG(2000)

The effect of DSPE-PEG(2000) on the structure of a POPC/DDAB monolayer was investigated using the Langmuir method (Figure 2).

**Figure 2:**
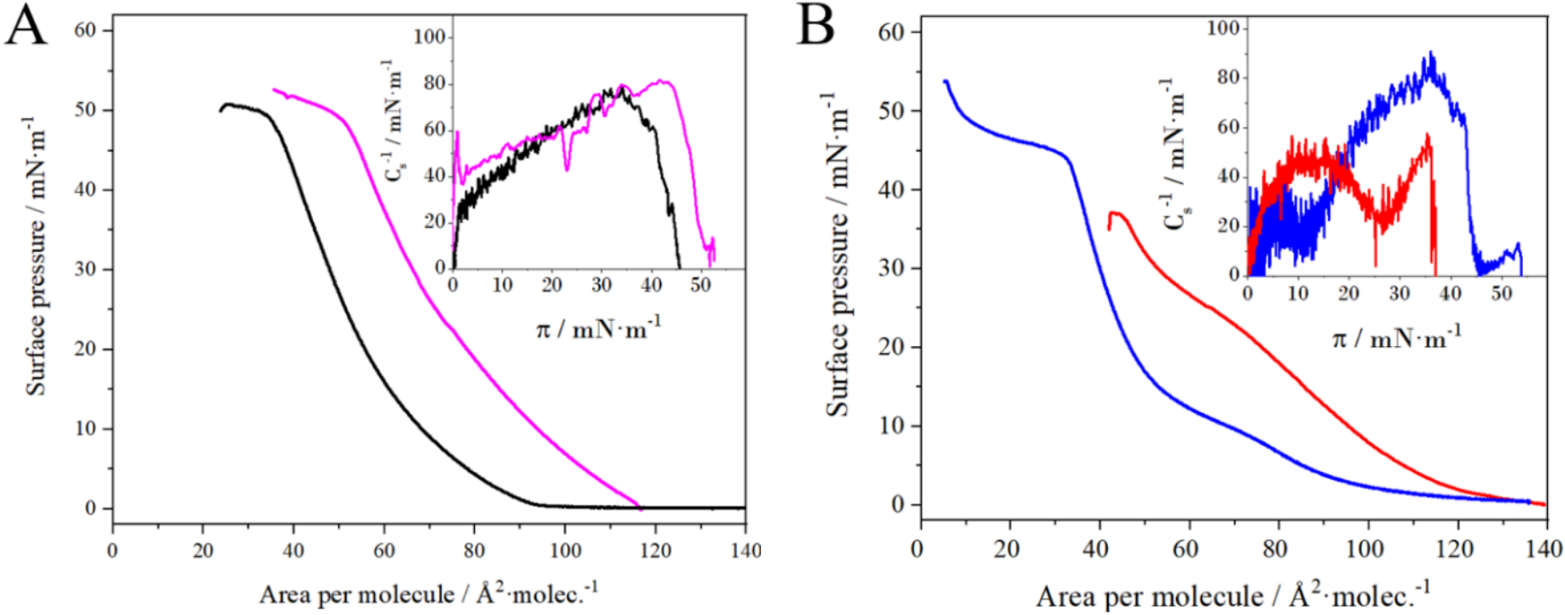
Surface pressure – area per molecule isotherms for (A) POPC/DDAB 9:1 (black) and (B) POPC/DDAB/DSPE-PEG(2000) 8:1.5:0.5 (blue) monolayers in the absence and presence of HSA 0.1% (B—pink, C—red). Insets: dependence of the compressibility modulus on surface pressure (*T* = 22 ± 1 °C).

Preliminary analysis of monolayers composed of individual components—POPC, DDAB, and DSPE-PEG(2000) (Figure S1A–C)—revealed the characteristics of the layers described below. POPC, which has a palmitic chain and a second singly unsaturated oleic chain, usually forms layers with medium packing degree and belongs to the expanded liquid phase (LC, *C*_smax_^–1^ = 70 mN/m).^25,31^ DDAB, a positively charged bromide salt, forms close-packed layers where the area per molecule in the well-packed layer is 41.1 Å^2^/molec. (Table S1). The other characteristic feature of this cationic lipid is the phase transition at a pressure of about 15 mN/m. In contrast, we also use the hydrophobic derivative of PEG, which is negatively charged—DSPE-PEG(2000). As a polymer with a large hydrophilic part, it organizes itself at the air/water interface, creating layers with a very high degree of fluidity (low maximum *C*_s_^–1^ value).^32^

Figure 2A shows the π – *A* isotherm for the POPC/DDAB 9/1 layer, corresponding to a single monolayer of the carrier. The maximum compressibility modulus of the POPC lipid and the mixture (∼90 mN/m), suggests that the lipids coexist in the form of an expanded liquid phase, but without the phase transition, characteristic for DDAB alone. This means that DDAB-POPC interactions prevent the system from phase transitions typical for the DDAB monolayer (Figure S1B). A similar effect of DDAB was previously noted for other mixed layers- DMPC/DDAB 3:1 and DPPC/DDAB 3:1.^33,34^

The addition of PEGylated lipid (DSPE-PEG(2000)) alters the shape of the π – *A* isotherm, indicating a reorganization in the layer structure (Figure 2B and Table 1). The presence of the polymer significantly affects the area per particle at lower pressure values, even though the polymer itself shows strong surface activity in this region.^32^ Part of DSPE is located inside the lipid layer, but it does not occupy too much area, as it has simple, long 18-carbon acyl chains and a polar head consisting of phosphatidylethanolamine (PE). The PEG chains of DSPE-PEG(2000) cause steric hindrance which affects the lipids packing in the monolayer. Balance charges in the mixture (positive for DDAB and negative for DSPE-PEG(2000)) reduce the influence of any electrostatic interactions on the layer structure. This is why more tightly packed layers are obtained with a higher maximum compressibility modulus than for a two-component monolayer (Table 1). The phase transition at ca. 7 mN/m indicates the condensation of polymer chains under the influence of their interactions with phosphatidylcholine.

**Table 1:** Characteristic Parameters for Mixed Monolayers Corresponding to One Leaflet of Liposome Carriers in the Absence and Presence of HSA (0.1%)

	*A*_π=10 mN m_^_–1_^/Å^2^ molec.^–1^	*A*_π=30 mN m_^_–1_^/Å^2^ molec.^–1^	*C*_smax_^–1^/mN m^–1^
POPC/DDAB 9:1	69.8 ± 1.7	47.6 ± 1.3	66 ± 6
POPC/DDAB/DSPE-PEG(2000) 8.5:1:0.5	68.6 ± 0.7	39.5 ± 0.8	92 ± 10
POPC/DDAB 9:1 + HSA	93.0 ± 3.0	66.9 ± 2.2	82 ± 8
POPC/DDAB/DSPE-PEG(2000) 8.5:1:0.5 + HSA	95.4 ± 4.0	51.9 ± 1.2	54 ± 7

The major advantages of the systems employed in this study are the attractive, or less repulsive, interactions between the components in both layers compared to an ideally miscible layer, which clearly enhance the stability of the tested system. The A^Exc^ values, determined from eq 4 for the investigated system, are equal to −10.0 Å^2^ molec.^–1^ at π = 10 mN/m and −4.9 Å^2^ molec.^–1^ at π = 30 mN/m for the two-component layer. For the ternary layer, these values are even lower (Figure 3, blue), indicating that the presence of DSPE-PEG(2000) facilitates the mixing of components in the layer. This should be particularly advantageous for liposomes composed of these layers when they serve as drug carriers, as it results in a more compact and well-organized system. Better condensation favored the ternary layers for the carrier’s lipid envelope (Figure 2B). The presence of PEGylated lipid enhances miscibility and condensation because the PEG chains promote favorable interactions between molecules (electrostatic forces and/or hydrogen bonding), while in the POPC:DDAB system without DSPE-PEG(2000), such favorable interactions do not occur. The more thermodynamically stable layers are the three-component ones (–Δ*G^M^*_POPC/DDAB_ > −Δ*G^M^*_POPC/DDAB/DSPE‑PEG(2000)_). The value of %*A* exceeds that for the two-component monolayer almost twice (Figure 3B).

**Figure 3:**
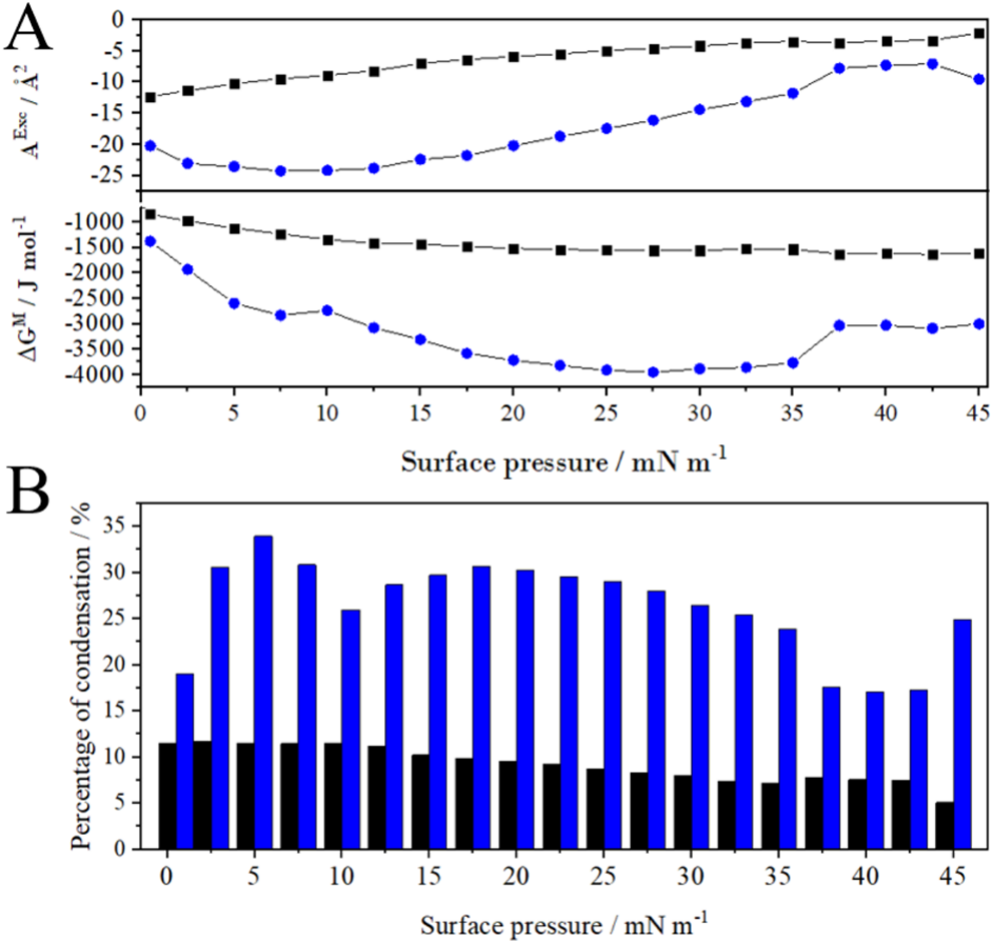
Thermodynamic parameters reflecting the interactions between components in mixed layers. (A) Excess area and mixing free energy and (B) percentage of condensation according to surface pressure values for POPC/DDAB 9:1 (black) and POPC/DDAB/DSPE-PEG(2000) 8:1.5:0.5 (blue).

### 3.2 Stability of POPC/DDAB and POPC/DDAB/DSPE-PEG(2000) Monolayers Spread over HSA/PBS Solution as the Subphase

Langmuir isotherms for mixed lipid layers spread in a subphase containing 0.1% HSA (Figure 2B) showed that the albumin present in the subphase strongly interacts with the two and three components, increasing the area per molecule by 19 and 12 Å^2^/molec., respectively. Interestingly, the elastic properties of the two-component layer remain practically unchanged (changes in *C*_s_^–1^ within the error range), while the degree of fluidity of the three-component layer increased significantly in the presence of HSA. It should be noted that HSA is also surface active and can self-organize at the air/water interface alone, as shown during barrier compression (Figure S2A,B). When various concentrations of HSA solutions were injected into the PBS buffer subphase, the surface pressure increased compared to that of the pure buffer solution. The penetration of HSA into the layers was easily seen in the compression/expansion cycles of the monolayers (Figure 4).

**Figure 4:**
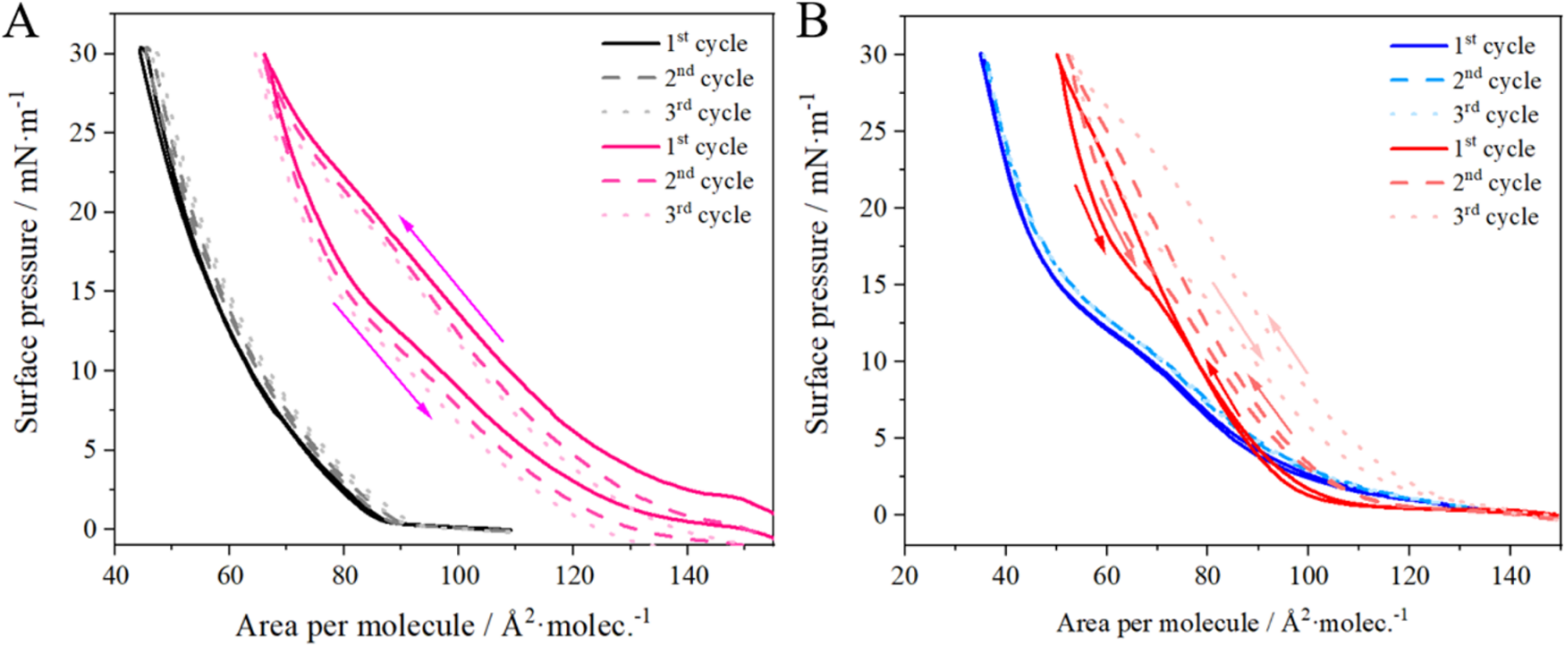
Compression – expansion cycles for monolayers (A) POPC/DDAB 9:1 and (B) POPC/DDAB/DSPE-PEG(2000) 8.5:1:0.5 compressed to 30 mN/m without (A —black, B–blue) and with HSA (A—pink, B—red) (*T* = 22 ± 1 °C).

According to the hysteresis theory for mixed layers,^25^ if the value of the thermodynamic parameters is close to 0 (no hysteresis loop), no irreversible domains, aggregates, or crystallization occur. For the systems analyzed, the free energy value closest to 0 was observed when the layers were not exposed to albumin (Table S2). For the ternary layer, perfectly retraceable cycles are observed (Figure 4B), and for the bicomponent layer, shifts of subsequent cycles were also almost negligible (Figure 4A). Negative values of the thermodynamic parameters indicate cohesive forces, and in the case of the ternary layer the formation of aggregates was observed after exposure to albumin (*T*Δ*S* ^hys^ < 0). The lower values of Δ*H* ^*hys*^ confirm that HSA binds strongly to the positively charged layers represented here by the POPC/DDAB system.^35^ Indeed, the DSPE-PEG(2000) component neutralizes the charge of DDAB in the layer; however, interactions with albumin are observed, leading to the formation of irreversible structures, which in turn result in an increased surface area per molecule with each subsequent cycle.

From the point of view of the circulation of carriers in the bloodstream, it is more relevant to monitor the effect of HSA on the preformed layers. Therefore, the monolayers POPC/DDAB and POPC/DDAB/DSPE-PEG(2000) were first compressed to a surface pressure of 30 mN/m (which is assumed to correspond to that of the biological membranes) and left on the PBS buffer subphase containing 0.1% HSA for 90 min (Figure 5). The results for individual lipid components presented in the Supporting Information (Figure S3) allow us to compare the extent of interaction with those found for the mixed lipid layers.

**Figure 5:**
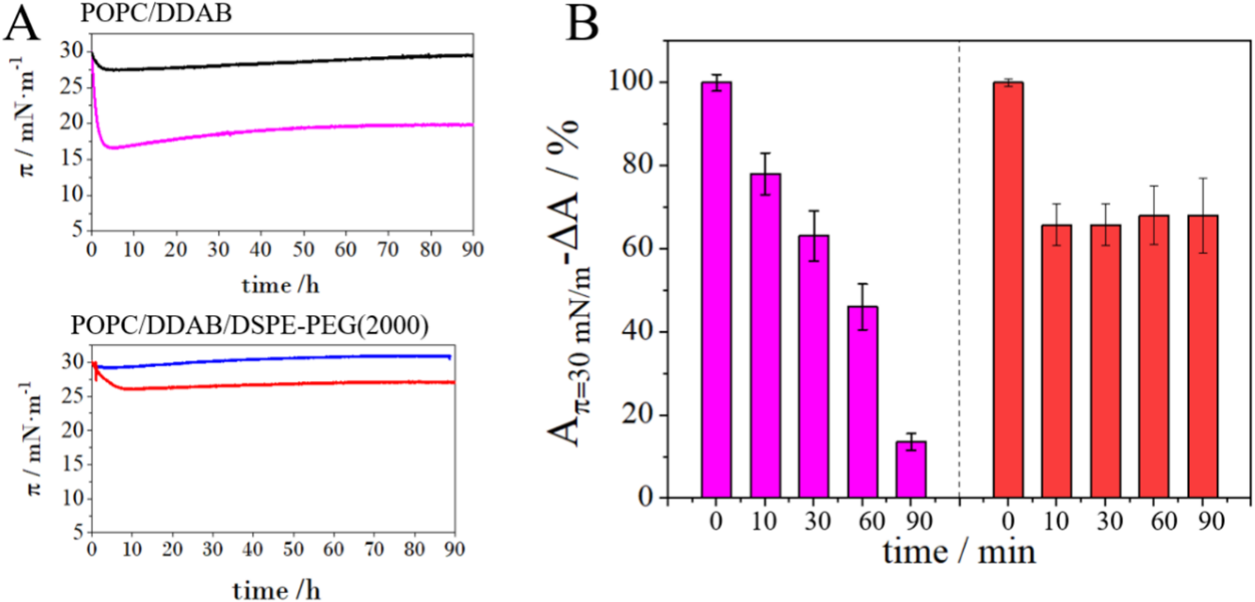
(A) Changes in the stability of the layer monitored by surface pressure over time dependences for mixed monolayers of POPC/DDAB 9:1 (black) and POPC/DDAB/DSPE-PEG(2000) 8:1.5:0.5 (blue) in absence and in the presence of 0.1% HSA (A—pink and B—red). (B) Changes in the area per molecule over time after HSA injection (0.1%) relative to the value of the area per molecule over time for the monolayer not exposed to albumin at 30 mN/m. Monolayers were previously compressed to the desired surface pressure, and the surface area was kept constant during the measurements.

The ternary monolayer is more stable than the two-component one, which correlates with excess parameters (*A^Exc^*_POPC/DDAB_ < *A* ^Exc^_POPC/DDAB/DSPE‑PEG(2000)_). Injection of serum albumin at a final concentration of 0.1% into the subphase further affects the two-component layer. In the first 10 min, there is a decrease in the surface pressure value occurs; after that, the layer stabilizes at a pressure of 17 mN/m. A continuous decrease in the area per molecule (Δ*A*) over time when the surface pressure of 30 mN/m is maintained. Importantly, the HSA effect is much weaker for the system containing DSPE-PEG(2000) (Table 1 and Figure 5B). PEGylated lipid residues protect the monolayer from the influence of albumin.

Changes in the orientation of functional groups in monolayers under the influence of albumin were monitored using PM-IRRAS (Figure 6 and Table 2). Albumin was injected into the subphase at the moment when the monolayer at the air–water interface was compressed to 30 mN/m, to check the formation of an HSA film on the surface of the lipid layer. The main regions containing characteristic bands of the primary component, POPC, were analyzed. In the spectrum of the main component (POPC), two regions can be distinguished: the ester group region of ester groups above 1700 cm^–1^ and the second region connected to the phosphate groups in the range 1300 to 1000 cm^–1^.

**Figure 6:**
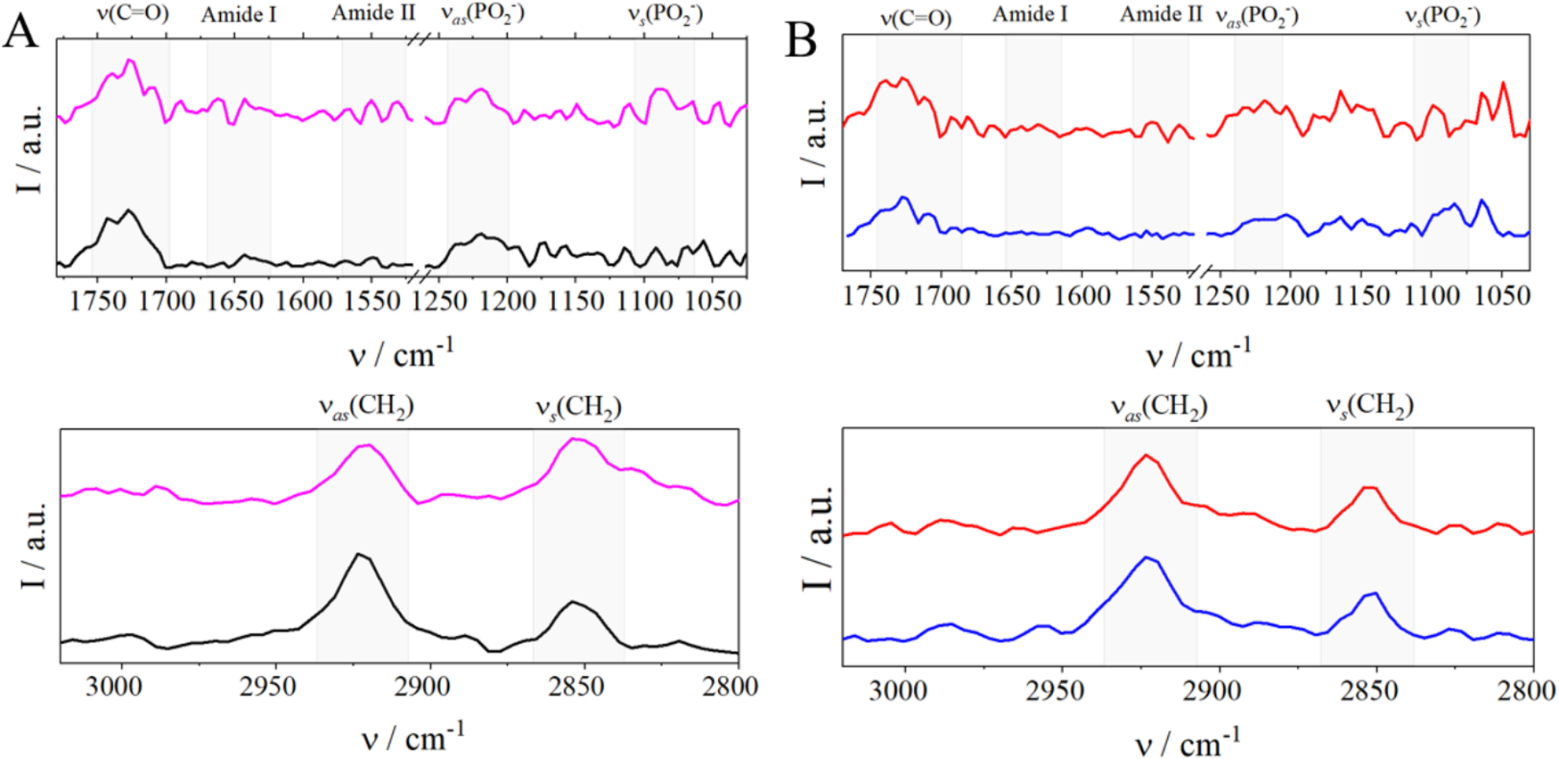
PM-IRRAS spectra in the 1800 to 1000 cm^–1^ (upper panel) and 3050 to 2750 cm^–1^ (bottom panel) regions of (A) POPC/DDAB 9:1 and (B) POPC/DDAB/DSPE-PEG(2000) 8.5:1:0.5 monolayers compressed to 30 mN/m without (black and blue) and with HSA (pink and red).

**Table 2:** PM-IRRAS Band Position (in cm^–1^) for POPC/DDAB 9:1 and POPC/DDAB/DSPE-PEG(2000) 8.5:1:0.5 Monolayers Compressed to 30 mN/m Without and With HSA (0.1%)

subphase	ν_as_(CH_2_)	ν_s_(CH_2_)	ν(C = O)	amide I	amide II	ν_as_(PO_2_^_–_^)	ν_s_(PO_2_^_–_^)	ν_as_C–O[P]
	POPC/DDAB 9:1							
PBS	2924	2954	1743; 1728	-	-	1219	1091	1059
+HSA 0.1%	2919	2850	1739; 1728	1662; 1643	1550; 1531	1219	1087	1064
	POPC/DDAB/DSPE-PEG(2000) 8.5:1:0.5							
PBS	2924	2854	1743; 1728	-	-	1219	1091	1064
+HSA 0.1%	2924	2854	1739; 1728	1662; 1643	-	1219; 1234	1099	1064

The characteristic bands for POPC/DDAB at wavelengths of 2924 and 2850 cm^–1^ are the asymmetric and symmetric vibrations of the CH_2_ groups of the POPC acyl chains, respectively.^36^ The bands assigned to scissoring H–C–H bending (1462 cm^–1^) are not well developed, therefore this fragment of the spectrum was omitted. The bands due to the vibrations of CH_2_ groups of the DSPE-PEG(2000) molecules are seen at the same wavelengths, and the low-intensity C = O and C–O bands appear at ∼ 1740 and ∼ 1060 cm^–1^, respectively.^37^ The presence of DSPE-PEG(2000) affects only the asymmetric vibrations in the C–O­[P] bond, and this change is small, as mentioned above. Due to the lack of visible changes in the position of the bands caused by symmetric and asymmetric vibrations of CH_2_ bonds in the fatty acid chains (in the hydrophobic part of the layer), we suggest the absence of major changes in the orientation of these functional groups after the introduction of DSPE-PEG(2000) into the system (Table 2).

The most intense bands in the spectrum are those related to ν­(C=O), corresponding to the formation of hydrogen bonds and the presence of double bonds in the fatty acid residues.

Human albumin reduces shifts the band originating from the unhydrated C=O bond to a wavelength of approximately 1739 cm^–1^. The presence of albumin is also revealed by the presence of the Amide I and Amide II bands at wavelengths of 1662, 1643 cm^–1^ and 1550, 1531 cm^–1^, respectively. Interestingly, the presence of 0.1% HSA also impacted the fluidity of the bicomponent layer and led to a shift of the ν_as_(CH_2_) and ν_s_(CH_2_) bands toward lower wavelengths. It indicates increased rigidity and lower mobility of the chains due to the incorporation of protein between them, which is consistent with the increasing compression modulus calculated from the isotherms (Figure 2B).

Although it was proven that HSA penetrates the three-component POPC/DDAB/DSPE-PEG(2000) layer, its presence introduces only negligible changes in the spectral characteristics of the tested layer. A better separation of the bands for asymmetric vibrations from the PO_2_^–^ bond was observed (visible bands for 1234 and 1219 cm^–1^) and a shift of the band from symmetric vibrations for this functional group to 1099 cm^–1^. The band appearing at a wavelength of about 1203–1206 cm^–1^ corresponds to stretching C–N bonds,^36^ but they appear only as shoulders to the more intense bands corresponding to the vibrations of PO_2_^–^ groups. Even strongly interacting albumin is barely visible in the spectrum recorded half an hour after its introduction into the system—in this case, only the Amide I bands at approximately 1662 and 1643 cm^–1^ were detected. No significant shifts in the Amide I band were observed in the presence of hydrophilic PEG chains, suggesting that albumin does not undergo structural rearrangements upon interaction, supporting the hypothesis that these chains act only as a steric or energetic barrier. This is consistent with the results of monolayer experiments, which show better stability of the surface pressure and area per molecule over time, also in the presence of HSA for the three-component layers. Therefore, it can be concluded that strong hydrophilic chains protruding from the layer form a barrier for albumin and that they can access only the hydrophilic part without entering the hydrophobic chain region of the monolayer.

### 3.3 Characteristics of POPC/DDAB and POPC/DDAB/DSPE-PEG(2000) Liposomes

The investigations presented so far showed the utility of the monolayer method in describing the specific interactions of the lipid layers with HSA and the advantages of using the POPC/DDAB/DSPE-PEG(2000) system over the POPC/DDAB—based one. Knowing this, we could prepare, characterize, and use liposomes to host iguratimod. An important aspect of the production of liposome drug carriers is their size. The range of 50 to 200 nm (SUV, LUV) was assumed to be the most suitable in terms of stability and half-life of circulation in the body (Figures 7 and S4).^38^ Extrusion through 100 nm diameter pores was followed by incubation for 2 h in 1% HSA solution at 36.6 °C. Figure S4 and Table S3 show the PDI values are quite low (<0.3), showing that the obtained liposomes have very similar sizes and acceptable homogeneity, useful for biological and pharmaceutical applications.

**Figure 7:**
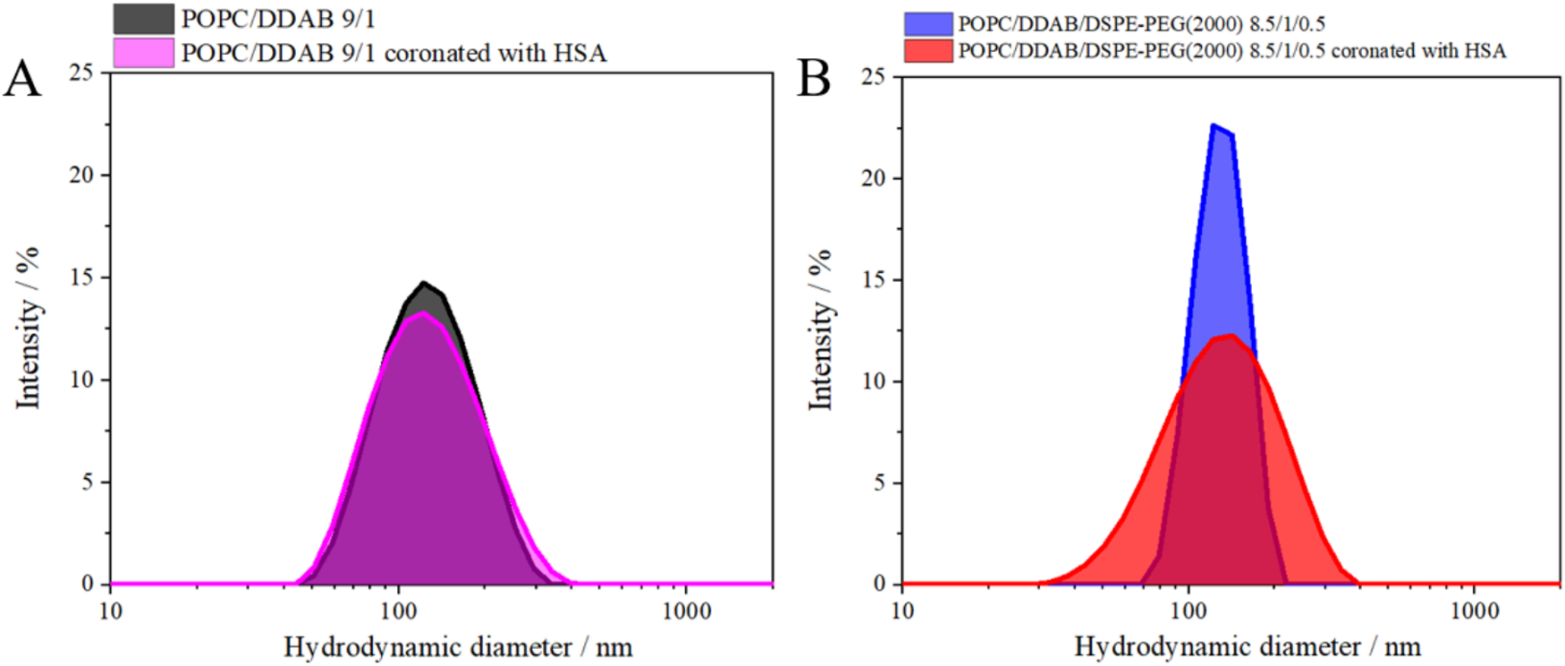
Histograms showing the hydrodynamic diameter of the studied systems (A) POPC/DDAB 9/1 and (B) POPC/DDAB/DSPE-PEG(2000) 8/1.5/0.5 resulting from a Gaussian fit to the intensity versus diameter curves. The measurement temperature is 36.6 °C.

Corona formation is reflected in the increase in the hydrodynamic diameter and the changes in the zeta potential upon incubation in the HSA solution. A slightly larger value of the hydrodynamic diameter was found for the PEGylated structures. Therefore, we can conclude that the hydrophilic part of PEG forms an additional film surrounding the liposome, even at a content such as that present in the carriers we propose. The zeta values (Tables 3 and S3) confirm that the liposomes remain stable, as they oscillate around values of about −8 mV for POPC/DDAB and about 0 mV for POPC/DDAB/DSPE-PEG(2000) liposomes. The negative zeta potential value for liposomes composed of zwitterions, as in the case of DPPC or DOPC (in phosphate buffer), was repeatedly reported.^39^ Incubation in 1% HSA changes the zeta value to more positive by approximately. 8.5 mV. The curvature of the two-component lipid bilayer, which we calculate based on the hydrodynamic diameter (*C =* 2/*D*_h_), influences the interactions between lipids and the protein in that adsorption of corona protein is favored for larger liposomes. This is supported by our data for two-component liposomes. Larger POPC/DDAB (9:1) liposomes with smaller curvature show more than a 2-fold increase in hydrodynamic diameter after incubation with proteins compared to smaller liposomes with bigger curvature. (Table S3 and Figure S5)

**Table 3:** Parameters Characterizing the Drug Carriers Studied

	in the absence of HSA		in the presence of HSA	
	hydrodynamic diameter^PDI^/nm	zeta potential/mV	hydrodynamic diameter^PDI^/nm	zeta potential/mV
POPC/DDAB 9:1	110^0.142^ ± 9	–7.55 ± 0.4	135^0.152^ ± 5	8.99 ± 0.20
POPC/DDAB/DSPE-PEG(2000) 8.5:1:0.5	130^0.098^ ± 7	0.043 ± 0.005	136^0.160^ ± 7	8.61 ± 0.19

However, it is important to note that not only the size and curvature, but also the membrane composition significantly affects the interactions with the protein corona. Therefore, both geometry (curvature) and chemical (lipid composition) properties of the nanostructures should be always considered in the interpretation of the results. (Table S4)

### 3.4 Drug Release Profiles: Effects of PEGylation and HSA Corona Protein

The properties of the liposomes, with the cargo, the semihydrophilic drug iguratimod, are presented in Figure S1 and Table S1. Upon analysis of the area per molecule, it is evident that the drug interacts only weakly with the POPC/DDAB/DSPE-PEG (2000) monolayer 8.5:1:0.5, while it is strongly incorporated into the two-component layer (Figure S1). This effect may be attributed to the more fluid nature of the two-component layer, making it easier for the drug to penetrate the layer. The drug does not influence the elastic properties of the layers themselves, as evidenced by the consistent maximum values of the compressibility modulus (Table S1). Despite the relatively low fraction of DDAB lipid, its inclusion in the mixture plays a significant role in enhancing the interactions between the drug and the POPC/DDAB layer (see Supporting Information Figure S1 and Table S1). However, a DSPE-PEG lipid is a barrier component that ″blocks″ the drug’s interactions with the structure of the membrane. The IR spectra at the air/water interface reflect slight changes of the monolayers due to the presence of drug. (Figure S6) For both POPC/DDAB 9/1 and POPC/DDAB/DSPE-PEG(2000) 8.5:1:0.5 layers, the drug affects the ordering of the monolayer structure, making it slightly more fluid, as evidenced by the shift of the asymmetric CH_2_ stretching band from 2924 to 2920 cm^–1^, while no observable effect on the symmetric vibrations was noted. Additionally, interactions in the hydrophilic region are mostly around the phosphate groups (PO_2_^–^), where a so-called blue shift is observed, suggesting a reduction in the hydration of these groups due to drug binding. Weaker interactions of iguratimod (even at high concentrations) with the ternary layer indicate that the drug will not accumulate on the surface of the lipid layer during encapsulation. Both types of liposomes, after hydration with an iguratimod solution at a concentration of 10^–5^ M, did not show significant changes in the hydrodynamic diameter for extruded liposomes. Due to the degree of lipophilicity of iguratimod (logP = 2.14), its interactions with the hydrophilic outer layer of the liposome should be considered - interactions with the POPC and DDAB polar heads, which were demonstrated in measurements using the Langmuir method (Figure S1A). Drugs with logP in the range of 1 to 3 can interact with both the hydrophilic and hydrophobic parts of the particles. The above studies allowed us to exclude the formation of a drug coat around the carrier, ruling out earlier speculations about the drug-membrane component interaction. The addition of the drug does not affect the charges of the liposomes, since in PBS buffer at pH 7.4, the neutral form of Igu predominates (p*K*_a_ 2.96).^40^ The zeta potential values closer to 0 for ternary systems indicate their greater stability compared to binary liposomes. Thus, assuming that the drug is incorporated into the inner part of the liposomes (aqueous core), the presence of Igu in the system does not affect the formation of the HSA corona protein, as indicated by the same sizes (within the margin of error) of the coronated structures. The above studies also rule out the behavior of iguratimod as an interface-associated drug, as suggested by its semihydrophilic structure. The most important point in drug carrier research is their ability to encapsulate a therapeutic agent. In these studies, a method was used to separate liposomes with encapsulated drug from unbound drug on a chromatographic column filled with Sephadex G-25. A huge advantage of the noninvasive method was the possibility of preserving fractions of separated liposomes and subjecting them to further tests and controlled drug release. A calibration curve for the drug used was recorded (Figure S6) and used to determine encapsulation efficiency (EE%).

The characteristic absorption band for iguratimod is 258 nm,^40^ and the linear equation is *y* = 5251*x* + 0.012. The encapsulation efficiency is 83% (extruded) and 82% (nonextruded) for the POPC/DDAB 9/1 sample, and for POPC/DDAB/DSPE-PEG(2000) 8.5:1:0.5, the EE% is equal to 91% (extruded) and 90% (nonextruded).^37^ Extrusion did not significantly affect the degree of encapsulation of the drug, which is good news because of the lack of loss of the therapeutic agent in the carrier preparation process.

Two mathematical models were applied to analyze drug release from liposomal carriers: the Higuchi model for the initial release phase and the Korsmeyer-Peppas model (K–P) for the complete release profile (Table S5). Drug release studies allowed for the determination of the effect of PEGylation and the HSA protein. Measurements were made in PBS buffer, pH 7.4, and at a constant temperature of 36.6 °C. For non-HSA-incubated liposomes, after 200 min, the drug release percentage was approximately 25%, while for HSA-coated structures it is approximately 33%. The proposed mechanism is that HSA binding may induce slight reorganization of the outer leaflet of two-component liposomes, increasing membrane porosity and facilitating drug diffusion.^7,24,41^ Although iguratimod is amphiphilic, albumin can still enhance its solubility by forming transient complexes, as it commonly does with hydrophobic compounds. Furthermore, HSA appears to gradually increase liposomal permeability, a phenomenon confirmed in the three-component system, where surface alterations were less pronounced (e.g., Figures 5 and S4). These combined effects likely contribute to the enhanced drug release kinetics observed in the presence of albumin.

In each of the investigated cases, a gradual release of the drug is observed in 1 h, followed by stabilization (plateau state) (Figure 8). The release of the drug from the three component layers is slower and the corona protein causes the percentage of drug release within 200 min to be 5% lower.

**Figure 8:**
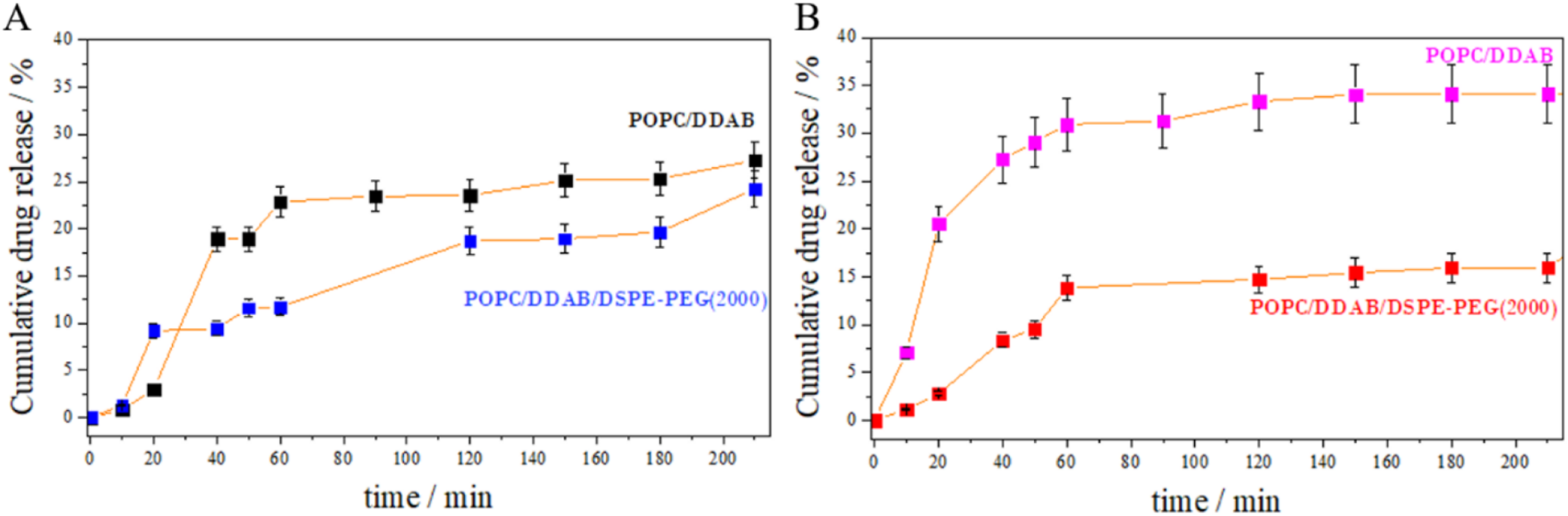
Release profiles of iguratimod from POPC/DDAB 9:1 and POPC/DDAB/DSPE-PEG(2000) 8:1.5:0.5 lipid carriers (A) without and (B) with the HSA corona in a buffer environment of PBS (pH = 7.4) at 36.6 °C.

For POPC/DDAB (9:1) liposomes, uncoated and coated with human serum albumin (HSA), the Higuchi model showed a good fit (R^2^ = 0.949 and 0.975, respectively), indicating diffusion-driven release (see also Figure S7). The K–P model applied to the full release data supported a non-Fickian anomalous diffusion mechanism, possibly involving matrix swelling. The release exponent (*n* = 0.98) for uncoated liposomes indicated uncontrolled diffusion, possibly due to structural destabilization after the release of iguratimod. On the contrary, PEGylated liposomes showed a reduced burst release and a lower *n* value (0.78), also consistent with anomalous diffusion. The fit of the Higuchi model (*R* ^2^ = 0.842) was worse, although cumulative release vs square root of time remained linear. PEGylation likely increased hydrophilicity and introduced diffusion resistance, leading to more sustained release. The improved fit to the K–P model (higher *R* ^2^) suggests that PEG also contributes to carrier stabilization.

In the presence of HSA corona, the PEGylated liposomes showed a release profile similar to that of the binary system. Although the linearity of the Higuchi model remained, the matrix effects linked to the PEG chains were apparent, by a more uniform drug release over time. All formulations exhibited an initial burst phase followed by a plateau, which may result from the amphiphilic nature of iguratimod and its complex interactions with lipids.

## 4 Conclusions

The Langmuir technique, which is a method for studying the behavior of monolayers at the air–water interface, was found here to be a powerful way to explore the surface properties of layers forming liposomes. It helps to understand the interactions between liposome components (POPC-DDAB-DSPE-PEG(2000)) and the encapsulated drug (iguratimod) and, more importantly, the impact of human serum albumin, one of the most abundant plasma proteins,^7^ on the lipid carrier. Three components with different surface properties were selected to design drug carrier models. POPC provides a high affinity for the carrier toward biological membranes. Positively charged surfactant, DDAB enhances the drug-envelope interaction, whereas PEGylated DSPE, with its hydrophilic alcohol chains, creates steric hindrance that affects both the drug and albumin. The surface properties of the two-component films revealed the interactions between POPC and DDAB, leading to a more stable and homogeneous layer. Incorporation of DSPE-PEG(2000) resulted in an even more compact monolayer with an increased maximum compressibility modulus, indicating improved structural stability (see Table 1). The phase transition observed at ∼ 7 mN/m, absent in the binary layer, suggests that PEGylation induces polymer chain condensation via interactions with phosphatidylcholine.

The excess parameters derived from the Langmuir experiments (*A* ^Exc^, Δ*G* ^M^) confirmed that DSPE-PEG(2000) stabilizes and integrates the lipid layers, to form stable liposomes. Hysteresis observed in the compression–decompression experiments supports such a conclusion, demonstrating that PEG residues also improve layer cohesion in the presence of albumin. 0.1% HSA led to a rapid decrease in the surface pressure of the binary system within the first 10 min, while the ternary system was clearly less affected, highlighting the protective role of PEG chains in minimizing corona protein-induced destabilization. The smaller extent of the penetration of the lipid membrane by HSA is also reflected in the smaller changes in the area per molecule (12 Å^2^/molecule) for the ternary POPC/DDAB/DSPE-PEG(2000) system.

The PMIRRAS measurements reveal that the introduction of HSA into the POPC/DDAB bicomponent system leads to a noticeable reduction in hydration, shifting the C=O band to ∼ 1739 cm^–1^, which also indicates increased layer rigidity. In contrast, the three-component POPC/DDAB/DSPE-PEG(2000) layer shows negligible changes in spectral characteristics and maintains better stability, with minimal shifts in the bands, confirming that the hydrophilic PEG chains create a barrier that limits the interaction of HSA with the hydrophobic lipid chains.

The binding of albumin to liposomes is proved by the increase in the hydrodynamic diameter and the more positive zeta potential of the nanoparticle. The presence of DSPE-PEG(2000) in the carrier envelope composition weakens the interaction with albumin, demonstrating a beneficial approach for both carrier stabilization and more sustainable release of the drug. Most importantly, ternary liposomes show higher drug encapsulation efficiency (91%), the drug release profile for these carriers under conditions close to physiological pH and temperature (36.6 °C) is more sustained. PEGylation reduced the kinetics of drug release of iguratimod by ∼ 5%, indicating better retention also in the presence of albumin. The drug release after 200 min was ∼ 33%.

Our research significantly improves the understanding of the properties and advantages of the POPC/DDAB/DSPE-PEG(2000) liposomes in corona protein environment. The above findings motivate further studies, including thorough investigations of the immune response to the presence of DSPE-PEG(2000) in the bloodstream, which would establish the utility of the proposed carrier in iguratimod-based therapy.

## Supplementary Material



## Notes

Surface pressure–area isotherms and compressibility modulus data for single and mixed lipid monolayers with and without iguratimod (Figure S1 and Table S1); isotherms and self-assembly behavior of HSA at the air/water interface (Figure S2), along with thermodynamic parameters of hysteresis for the two- and three component monolayers (Table S2); POPC, DDAB and DSPE-PEG(2000) monolayers stability over time in the presence and absence of HSA (Figure S3); hydrodynamic diameter distributions and characterization parameters of drug carriers before and after HSA corona formation (Figure S4 and S5 and Tables S3 and S4); IR spectra showing iguratimod effect (Figure S6 and Table S5); UV–vis absorbance spectra, calibration curve for iguratimod, and drug release modeling data (Figures S7 and S8 and Table S6) (PDF)
