# Correction to “Investigating Liposome Membrane Properties: Insights from Langmuir Monolayer Studies in the Corona Protein Environment”

**DOI:** 10.1021/acs.jpcb.5c06953

**Published:** 2025-10-28

**Authors:** Michalina Zaborowska-Mazurkiewicz, Natalia Kraśkiewicz, Piotr Sekuła, Renata Bilewicz

**Affiliations:** 49605 University of Warsaw, Faculty of Chemistry, Pasteura 1, 02093 Warsaw, Poland

**5:**
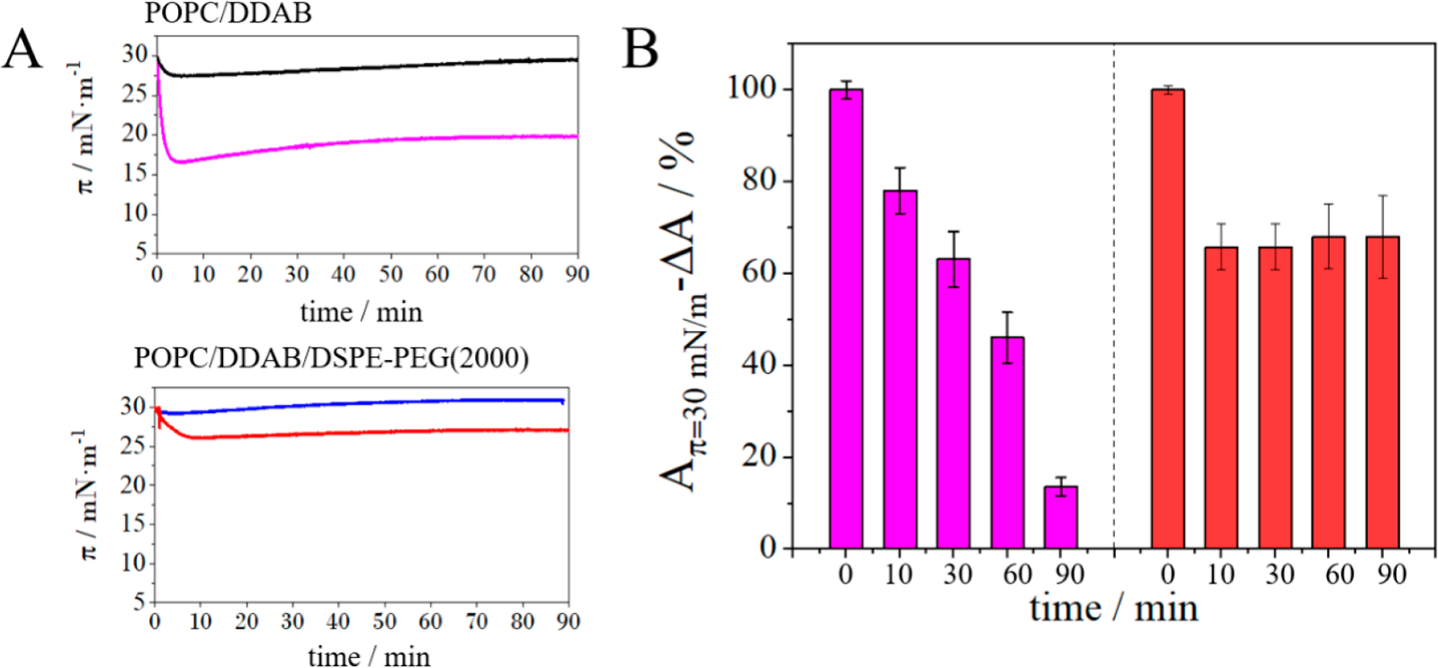
(A) Changes in the stability of the layer monitored by surface pressure over time (with constant area during measurement) dependences for mixed monolayers of POPC/DDAB 9/1 (black) and POPC/DDAB/DSPE-PEG(2000) 8/1.5/0.5 (blue) in the absence and in the presence of 0.1% HSA (A - pink and B - red). (B) Changes in the area per molecule over time (with constant surface pressure during measurement) after HSA injection (0.1%) relative to the value of the area per molecule over time for the monolayer not exposed to albumin at 30 mN/m.

