# Supplementary material for: Investigating Liposome Membrane Properties: Insights from Langmuir Monolayer Studies in the Corona Protein Environment
Source: J Phys Chem B. 2025 Aug 19;129(34):8742–53. doi: 10.1021/acs.jpcb.5c03676 (PMC12400416; doi:10.1021/acs.jpcb.5c03676)
Supplement: Supplementary file 1 [file jp5c03676_si_001.pdf]

Supporting materials

Title: Investigating liposome membrane properties: Insights from Langmuir monolayer studies in protein corona environment

Michalina Zaborowska-Mazurkiewicz, Natalia Kraśkiewicz, Piotr Sekuła, Renata Bilewicz*

University of Warsaw, Faculty of Chemistry, Pasteura 1, 02093 Warsaw, Poland

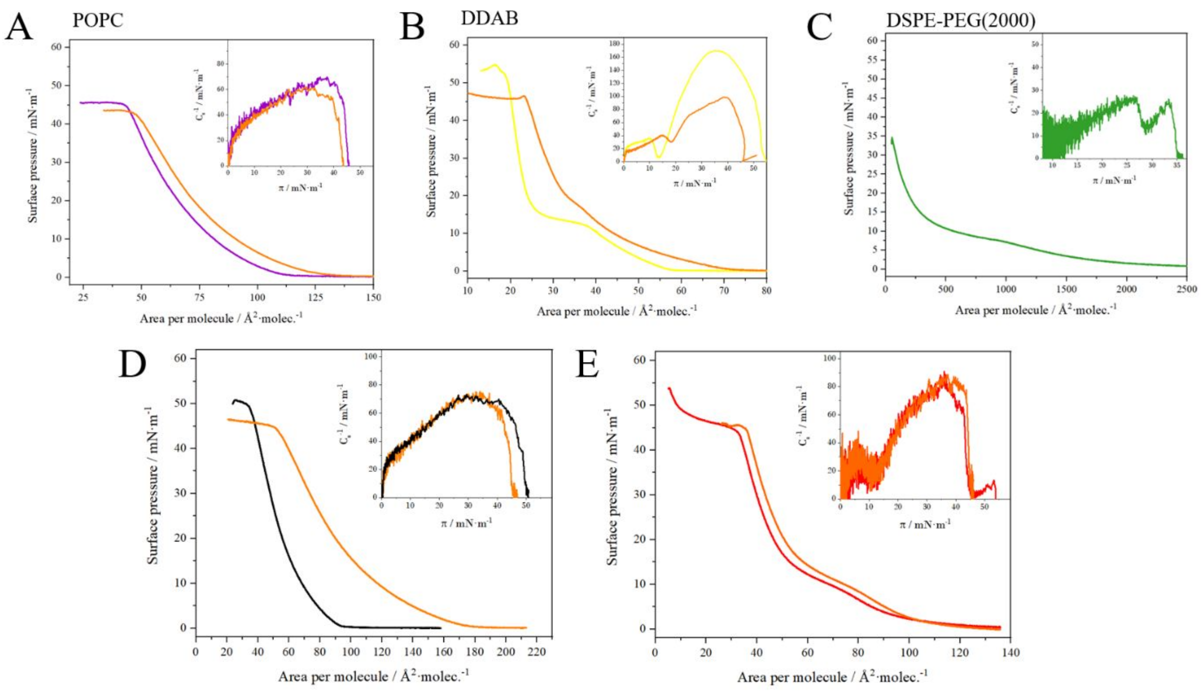

Figure S1 Surface pressure – area per molecule isotherms for monolayers with the compositions corresponding to that of the liposomes used single component monolayers of A) POPC, B) DDAB and C) DSPE-PEG2000; and mixed monolayers D) POPC/DDAB 9/1 (black) and E) POPC/DDAB/DSPE-PEG2000 8/1.5/0.5 (red) in the absence and presence of 10^{-5}M Iguratimod (orange). Insets: dependence of the compressibility modulus on the surface pressure (T = 22 ± 1°C).

Table S1 Characteristic parameters for single component monolayers forming drug carriers in the absence and presence of the Iguratimod

	$A_{\pi=10\ mM\ m^{-1}}$ / Å^2 molec.$^{-1}$	$A_{\pi=30\ mM\ m^{-1}}$ / Å^2 molec.$^{-1}$	$C_s^{-1}{}_{max}$ / mN m^{-1}

POPC	80.8 ± 0.7	55.8 ± 0.2	71 ± 4
POPC + Igu C=10^{-5} M	88.8 ± 1.7	58.8 ± 1.7	62 ± 1
DDAB	41.1 ± 0.6	22.8 ± 0.9	167 ± 14
DDAB + Igu C=10^{-5} M	43.2 ± 2.6	28.1 ± 2.0	108 ± 8
DPSE-PEG(2000)	412.0 ± 2.6	99.0 ± 1.9	28 ± 4
POPC/DDAB 9/1	69.8 ± 1.7	47.6 ± 1.3	66 ± 6
POPC/DDAB 9/1 + *Igu* C=10^{-5} M	114.4 ± 2.0	70.3 ± 1.2	74 ± 1
POPC/DDAB/DPSE-PEG(2000) 8.5/1/0.5	68.6 ± 0.7	39.5 ± 0.8	92 ± 10
POPC/DDAB/DPSE-PEG(2000) 8.5/1/0.5 + *Igu* C=10^{-5} M	73.4 ± 0.5	43.4 ± 0.2	78 ± 6

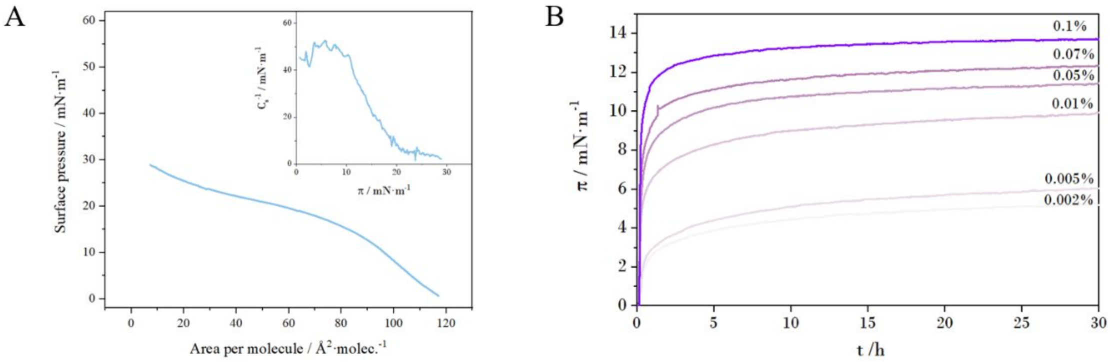

Figure S2 A) *Surface pressure – area per molecule isotherm for HSA 0.1 % layer. Insets: dependence of the compressibility modulus on the surface pressure (T = 22 ± 1°C). B) Self-assembly of human serum albumin (HSA) from the moment of injection of the protein solution into the subphase (unforced organization at the air/water interface).*

Table S2 Thermodynamic parameters based on the hysteresis of compression/expansion isotherms of POPC/DDAB 9/1 and POPC/DDAB/DSPE-PEG(2000) 8.5/1/0.5 monolayers .

Subphase		ΔG^{hys} / kcal· mol^{-1}	$T\Delta S^{hys}$ / kcal·K· mol^{-1}	ΔH^{hys} / kcal· mol^{-1}
		POPC/DDAB 9/1		
PBS	*1st cycle*	0.018±0.002	0.195±0.002	0.213±0.005
	2nd cycle	0.018±0.002	0.196±0.002	0.211±0.005
	3rd cycle	0.018±0.002	0.196±0.002	0.210±0.004
+ HSA 0.1%	*1st cycle*	-0.469±0.03	-1.87±0.02	-2.34±0.01
	2nd cycle	-0.414±0.03	-1.74±0.03	-2.15±0.03
	3rd cycle	-0.415±0.03	-1.73±0.03	-2.16±0.03
		POPC/DDAB/DSPE-PEG(2000) 8.5/1/0.5		
PBS	*1st cycle*	0.0167±0.001	0.105±0.005	0.121±0.05
	2nd cycle	0.0169±0.002	0.105±0.004	0.120±0.05
	3rd cycle	0.0169±0.001	0.100±0.005	0.122±0.05
+ HSA 0.1%	*1st cycle*	-0.080±0.005	-0.57±0.008	-0.65±0.04
	2nd cycle	-0.150±0.005	-0.85±0.008	-0.99±0.04
	3rd cycle	-0.048±0.005	-1.61±0.008	-1.66±0.04

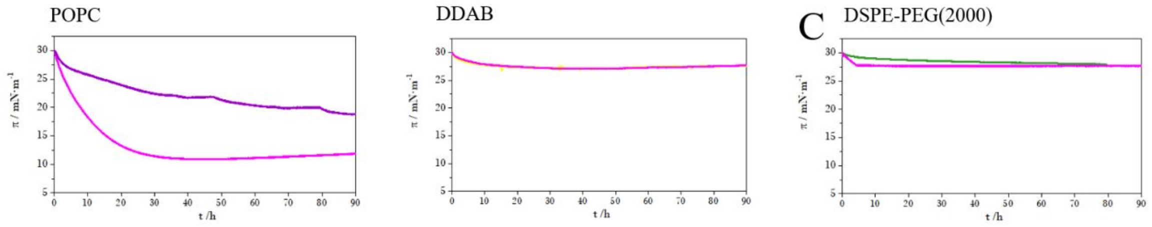

Figure S3 Changes in the stability of the layer monitored by the surface pressure over time dependences for mixed monolayers of POPC,DDAB and DSPE-PEG(2000) in the absence (A – pink) , and in the presence of 0.1%HSA (B - violet).

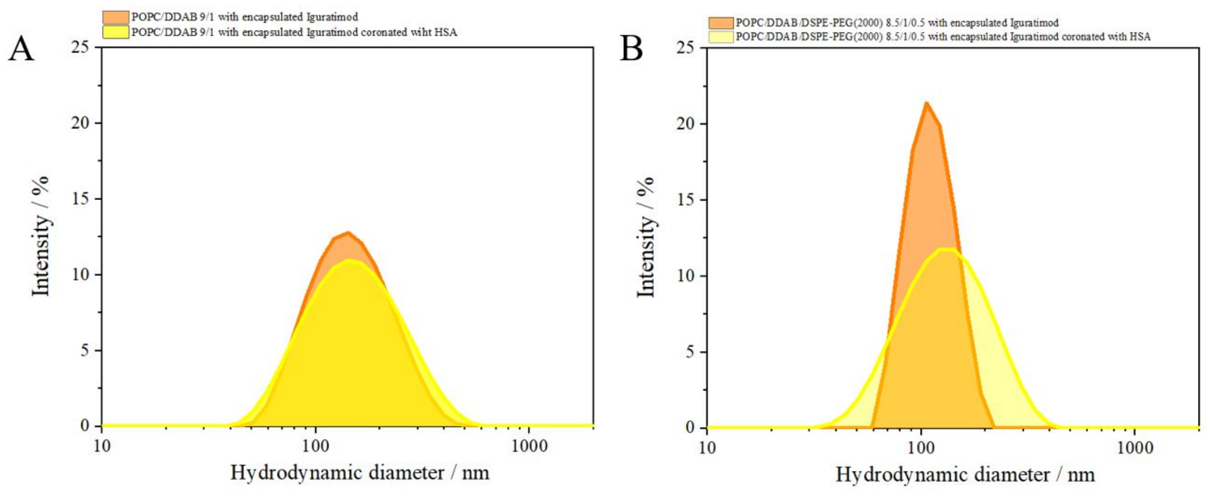

Figure S4 Histograms showing the hydrodynamic diameter of the studied systems with incorporated iguratimod: A) POPC/DDAB 9/1 and B) POPC/DDAB/DSPE-PEG(2000) 8/1.5/0.5 resulting from a Gaussian fit to the intensity versus diameter curves. The measurement temperature is 36.6°C.

Table S3: Parameters characterizing the drug carriers of two different sizes to show the dependence of HSA adsorption on liposome surface curvature.

POPC/DDAB 9/1 Liposomes of two different sizes	D_h / nm	C / nm^{-1}	D_h / nm	C / nm^{-1}
	Without HAS		With HSA	
Liposomes A (Larger curvature)	110± 9	0.018	135± 5	0.015
Liposomes B (Small curvature)	220± 11	0.0091	460± 20	0.0043

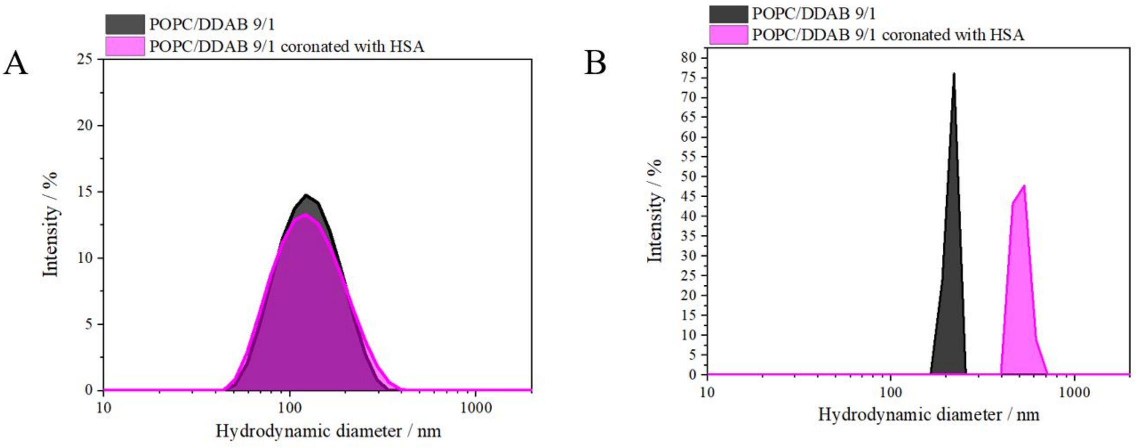

Figure S5: Histograms showing the hydrodynamic diameter of the studied systems (A – smaller, extruded and B – larger, non-extruded) POPC/DDAB 9/1 liposomes resulting from a Gaussian fit to the intensity versus diameter curves. The measurement temperature is 36.6 ° C.

Table S4 Parameters characterizing drug carriers

	Hydrodynamic diameter^{PDI} / nm – bare	Zeta potential / mV - bare	Hydrodynamic diameter^{PDI} / nm - HSA coronated	Zeta potential / mV – HSA coronated
POPC/:DDAB 9/1 + Igu C=10^{-5} M	$117^{0.211} \pm 5$	-7.85 ± 0.8	$137^{0.185} \pm 3$	8.51 ± 0.11
POPC:DDAB:DPSE-PEG2000 8.5/1/0.5 + Igu C=10^{-5} M	$117^{0.120} \pm 6$	1.810 ± 0.031	$141^{0.166} \pm 5$	8.81 ± 0.10

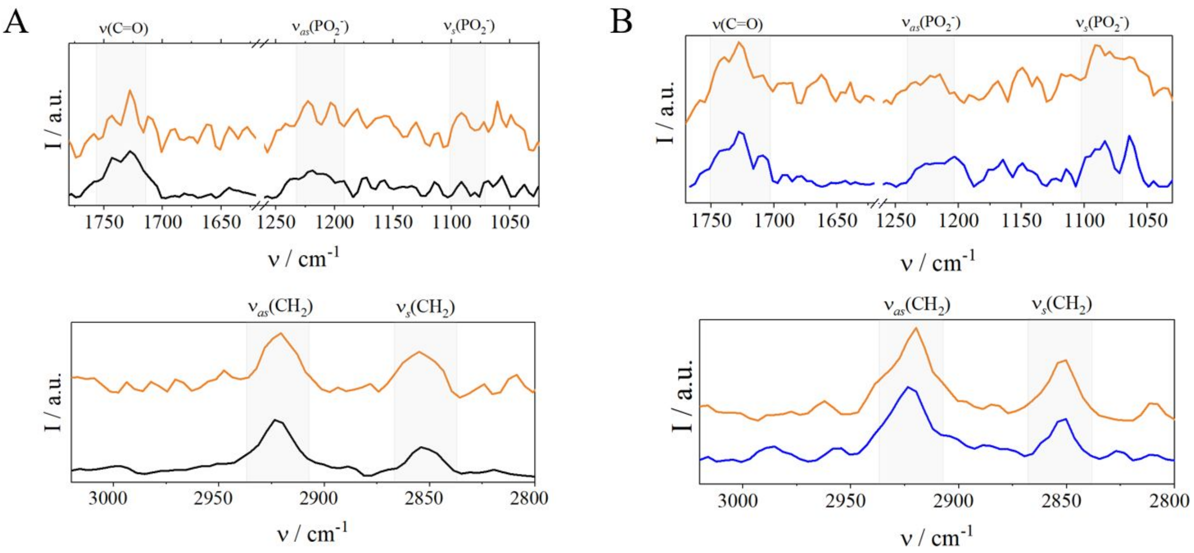

Figure S6 PM-IRRAS spectra in the 1800 cm^{-1} to 1000 cm^{-1} (upper panel) and 3050 cm^{-1} to 2750 cm^{-1} (bottom panel) regions of A) POPC/DDAB 9/1 and B) POPC/DDAB/DSPE-PEG(2000) 8.5/1/0.5 monolayers compressed to 30 mN/m without (black and blue) and with Igu (10^{-5} M) (orange).

Table S5 PM-IRRAS band position (in cm^{-1}) for POPC/DDAB 9/1 and POPC/DDAB/DSPE-PEG(2000) 8.5/1/0.5 monolayers compressed to 30 mN/m without and with iguratimod (10^{-5} M)

Subphase	$\nu_{as}(CH_2)$	$\nu_s(CH_2)$	$\nu(C=O)$	$\nu_{as}(PO_2^-)$	$\nu_s(PO_2^-)$	ν_{as}C-O[P]
			POPC/DDAB 9/1			
PBS	2924	2954	1743; 1728	1219	1091	1059
+ Igu 10^{-5} M	2920	2954	1743; 1728	1223	1091	1059
			POPC/DDAB/DSPE-PEG(2000) 8.5/1/0.5			
PBS	2924	2854	1743; 1728	1219	1091	1063
+ Igu 10^{-5} M	2920	2854	1739; 1724	1223	1091	-

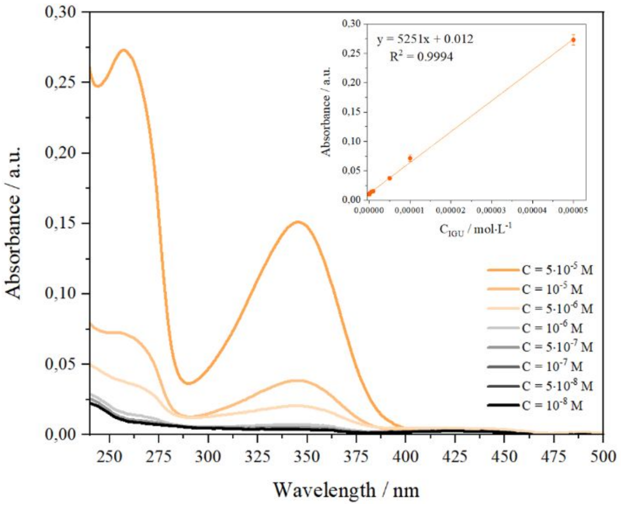

Figure S7 Absorbance - wavelength dependence for Iguratimod and calibration curve in the concentration range from 10^{-8} M to $5{\cdot}10^{-5}$ M.

Table S6 Tabulated equations representing the mathematical models of drug release from the studied liposomes

Mathematical model	*Zero order*	*First order*	*Higuchi*	*Korsmeyer - Peppas*
Time / min	POPC/DDAB 9/1			
0 – 60	Q = 8E-10t - 4E-09; **R^2 = 0.9507**	lnQ = 0.0604t - 20.132; R^2 = 0.8925	Q = 0.0119$t^{1/2}$ + 0.0256; **R^2 = 0.9507**	lnQ = 1.7714 lnt - 8.8559; **R^2 = 0.981**
60 – 210	Q = 1E-10t + 3E-08; R^2 = 0.897	lnQ = 0.0025t - 17.149; R^2 = **0.9316**	Q = 0.0018 $t^{1/2}$ + 0.0235; R^2 = 0.897	lnQ = 0.3665 lnt - 3.4802; R^2 = 0.852
0 – 210	Q = 3E-10t + 1E-08; R^2 = 0.7547	lnQ = 0.0107t - 18.368; R^2 = 0.4703	Q = 0.0038 $t^{1/2}$ + 0.0908; R^2 = 0.7547	lnQ = 0.9849 lnt - 6.3677; R^2 = 0.809
	POPC/DDAB 9/1 coronated with HSA			
0 – 60	Q = 7E-10t + 4E-09; R^2 = 0.9748	lnQ = 0.0437t – 19.603; R^2 = 0.9725	Q =1E-09 $t^{1/2}$ + 4E-09; **R^2 = 0.9748**	lnQ = 1.227 lnt - 7.0805; R^2 = 0.9829
60 – 210	Q = 5E-11t + 3E-08; R^2 = 0.9762	lnQ = 0.0013t – 17.16; R^2 = 0.9679	Q = 1E-09 $t^{1/2}$ + 4E-09; R^2 = 0.9762	lnQ = 0.1441 lnt - 2.5818; R^2 = 0.9216
0 – 210	Q = 2E-10t + 1E-08; R^2 = 0.7285	lnQ = 0.009t- 18.336; R^2 = 0.5618	Q = 4E-10 $t^{1/2}$ + 1E-08; R^2 = 0.7285	lnQ = 0.736 lnt - 5.529; **R^2 = 0.8765**
	POPC/DDAB/DSPE-PEG(2000) 8/1.5/0.5			
0 – 60	Q = 3E-10t - 5E-09; R^2 = 0.746	lnQ = 0.0278t - 19.132; R^2 = 0.6325	Q = 7E-10 $t^{1/2}$ + 3E-09; **R^2 = 0.8419**	lnQ = 0.8738 lnt - 6.2869; R^2 = 0.7953
60 – 210	Q = 2E-10t + 1E-08; R^2 = 0.9484	lnQ = 0.0048t - 17.844; R^2 = 0.9144	Q = 3E-10 $t^{1/2}$ + 1E-08; R^2 = 0.9484	lnQ = 0.6335 lnt - 5.4296; R^2 = 0.9704
0 – 210	Q = 2E-10t + 9E-09; **R^2 = 0.9521**	lnQ = 0.0079t - 18.405; R^2 = 0.703	Q = 4E-10 $t^{1/2}$ + 9E-09; **R^2 = 0.9521**	lnQ = 0.7019 lnt - 5.7406; **R^2 = 0.9053**
	POPC/DDAB/DSPE-PEG(2000) 8/1.5/0.5 coronated with HSA			
0 – 60	Q = 6E-10t - 2E-09; R^2 = 0.975	lnQ = 0.0436t - 19.603; R^2 = 0.9725	Q = 1E-9 $t^{1/2}$ + 4E-09; **R^2 = 0.9748**	lnQ = 1.2273 lnt - 7.3915; R^2 = 0.9829
60 – 210	Q = 1E-10t + 3E-08; R^2 = 0.6082	lnQ = 0.0022t - 17.294; R^2 = 0.9663	Q = 2E-10 $t^{1/2}$ + 3E-08; R^2 = 0.6282	lnQ = 0.2524 lnt - 3.389; R^2 = 0.525
0 – 210	Q = 2E-10t + 1E-08; R^2 = 0.833	lnQ = 0.0081t - 18.346; R^2 = 0.6116	Q = 4E-10 $t^{1/2}$ + 1E-08; R^2 = 0.8155	lnQ = 0.7574 lnt - 5.9023; **R^2 = 0.8899**

*Q is cumulative released drug (n_t/n)

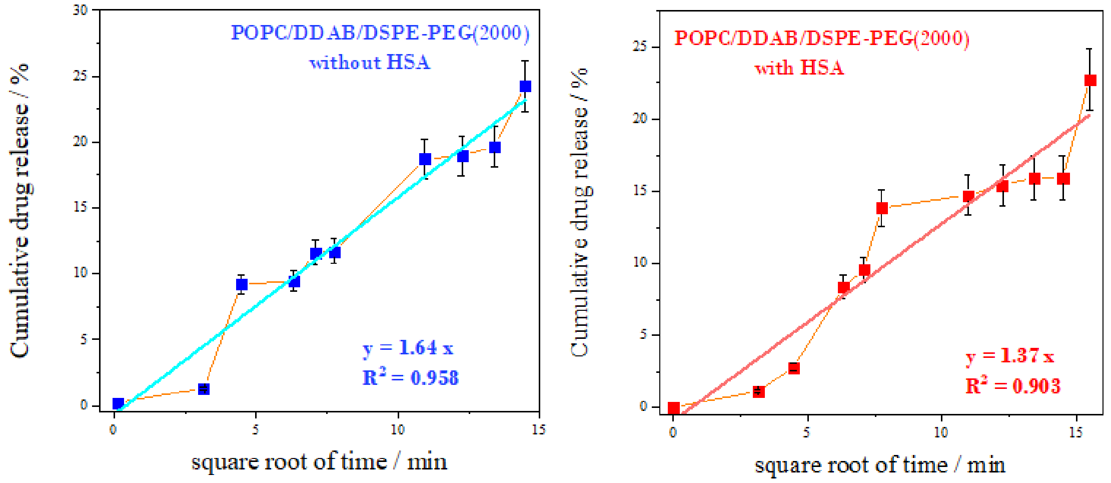

Figure S8 Cumulative drug release profiles versus the square root of time demonstrate linear fitting to the Higuchi model for iguratimod release from the ternary POPC/DDAB/DSPE-PEG(2000) liposomes, both in the absence and presence of a corona protein.
